# Comparative analysis of polydiphenylamine/MWCNT-COOH composites for supercapacitors: recovered *vs.* commercial nanotube electrodes

**DOI:** 10.1039/d5ra09202g

**Published:** 2026-03-04

**Authors:** C. S. Florica, A. Nila, M. Vaduva, C. Negrila, C. Bartha, Marouane Aannir, Ismael Saadoune, S. Bellucci, O. Cramariuc, M. Baibarac

**Affiliations:** a University of Bucharest, Faculty of Physics Atomistilor Street 405 Magurele Romania; b National Institute of Materials Physics, Laboratory of Optical Processes in Nanostructured Materials Atomistilor Street 405A Magurele Romania barac@infim.ro; c Mohammed VI Polytech Univ UM6P, ACER Lot 660, Hay Moulay Rachid Ben Guerir 43150 Morocco; d Nano Research Laboratory, Excellent Center, Baku State University Baku Azerbaijan; e Ecotec University Samborondón Ecuador; f IT Ctr Sci & Technol 25 Av Radu Beller Str Bucharest 011702 Romania

## Abstract

This paper reports a method for separating the constituents of a cathode containing a composite of the type polydiphenylamine (PDPA)/multi-walled carbon nanotube grafted with carboxyl groups (PDPA/MWCNT-COOH-1) as active material in end-of-life rechargeable lithium-ion batteries (RLIB) and their reuse as active materials in the field of other energy storage devices. The materials recovered from lithium battery cathodes are characterized by FTIR spectroscopy, Raman scattering, surface-enhanced Raman scattering (SERS), and X-ray diffraction. The use of the PDPA/MWCNT-COOH-1 composite as active material in the development of new energy storage devices, such as symmetric supercapacitors, is also reported. The performance comparison of the recycled composite material (PDPA/MWCNT-COOH-1 and MWCNT-COOH) with the one synthesized from pure chemical compounds (PDPA/MWCNT-COOH-2) is shown. Values of the capacitance of symmetrical supercapacitors, having as electrode materials the composites of the type of a mixture of PDPA/MWCNT-COOH-1 and MWCNT-COOH, as well as PDPA/MWCNT-COOH-2, are equal to 136.6 mF cm^−2^ and 112.59 mF cm^−2^. Increasing the concentration of the PDPA/MWCNT-COOH-2 active material in the electrode mass from 80 wt% to 84 wt% led to variations in the capacitance values of the symmetrical supercapacitors from 112.59 mF cm^−2^ to 145.5 mF cm^−2^.

## Abstract 2

This paper reports a method to separate the components of a cathode made of polydiphenylamine/carboxyl-grafted multi-wall carbon nanotube composite from rechargeable lithium-ion batteries and reuse them as active materials for supercapacitors.

## Graphical abstract

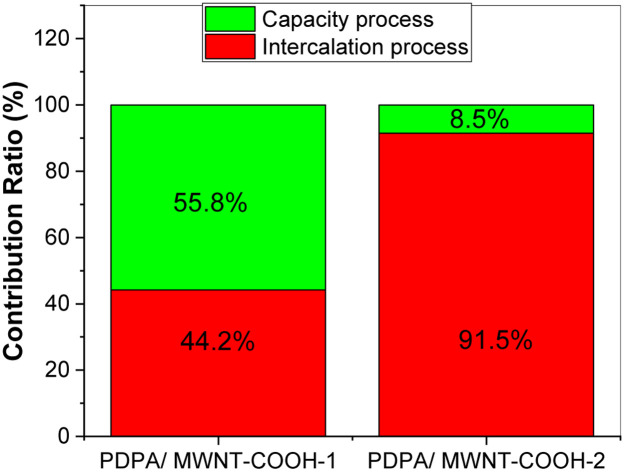

## 1. Introduction

The rapid expansion of the rechargeable Li-ion battery (RLIB) market has amplified potential risks in its upstream supply chain, which consists of many “critical materials” such as lithium, cobalt, nickel, and graphite.^1^ The growing need to mitigate resource scarcity and environmental impact has led to an increasing interest in recovering and reusing end-of-life (EoL) components to fabricate low-cost functional materials for new energy storage devices. Several recycling approaches have been developed for RLIBs, including: (i) the mechanical method, where electrode materials are separated from metallic and polymeric crushing, grinding and/or magnetic separation;^2^ (ii) the thermal treatment method, in which organic additives and binders are pyrolyzed at high temperatures to release the electrode materials from the foils;^3^ (iii) mechano-chemical approaches, using reactive media such as CaO to decompose poly(vinylidene fluoride) (PVDF) at moderate temperatures (≈300 °C) without HF release;^4^ (iv) the pyrometallurgical process, where pre-treated batteries are incinerated, converting metals (Co, Ni, Fe) into alloys while lithium remains in the slag fraction for later recovery;^5^ and (v) the hydrometallurgical process, which dissolves metals using acid or alkaline solutions for subsequent separation and purification.^6^ Recycling strategies increasingly target the valorization of electrode composites by: (a) employing spent battery powders in the oxidative polymerization of aniline and with recovered graphite, resulting in polyaniline/graphite and polypyrrole/graphite composites being obtained;^7,8^ and (b) recovery of single-walled carbon nanotubes (SWNTs) and their use in the generation of new functional groups in the presence of HNO_3_ accompanied by thermal oxidation in air or the interaction of SWNTs with HCl and thermal oxidation;^9^ and (c) PVDF decomposition from the spent RLIB cathode.^10,11^ Although there is an important advance in the use of electrode materials based on organic polymers, including conductive polymers, in RLIBs,^12,13^ to the best of our knowledge, little attention has been paid to the recovery and reuse of these materials and their composites with carbon nanostructures.

In this work, we address this gap by recovering the polydiphenylamine/multi-walled carbon nanotubes grafted with carboxyl groups (PDPA/MWCNT-COOH) composites from EoL RLIB cathodes and reusing them as active materials for supercapacitor electrodes. In this context, we note that PDPA has attracted increasing attention due to its favorable electrochemical stability and structural robustness.^14^ Compared with other conductive polymers such as polyaniline, PDPA exhibits reduced volumetric changes during repeated redox processes and improved cyclic stability, which are essential aspects for long-term electrochemical applications.^14^ In addition, the rigid aromatic structure of PDPA can contribute to a relatively wider electrochemical operating window and suppress degradation reactions, making it a suitable candidate as electrode active material in energy storage systems.^14^ Therefore, PDPA was selected in this work to make a trade-off between electrochemical performance and long-term stability. The separation of the PDPA/MWCNT-COOH composite was monitored using FTIR spectroscopy, Raman scattering, or surface-enhanced Raman scattering (SERS) and X-ray diffraction. Considering the increased interest in composites based on MWCNT functionalized with compounds containing amine groups, such as polyaniline, or conjugated mesoporous/microporous polymers for applications in the field of supercapacitors,^15–17^ the performances of the supercapacitors using the electrodes prepared from the recovered composite will be compared with the electrodes prepared from the commercial compounds. This work contributes to a better understanding of the separation of the constituents of cathodes of spent RLIB and opens new avenues for future research, which can aim to investigate in more deep how the active materials based on the PDPA/MWCNT-COOH composite recovered from cathodes of spent RLIB can be used in various applications.

## 2. Experiments

Compounds diphenylamine (DPA, ACS reagent grade ≥99%), multi-walled carbon nanotubes (MWCNTs, 98% carbon basis), multi-walled carbon nanotubes grafted with carboxylic groups (MWCNT-COOH, >90% carbon basis), potassium dichromate (K_2_Cr_2_O_7_, ACS reagent, ≥99.0%), sulfuric acid (H_2_SO_4_, ACS reagent, 95.0–98.0%), acetonitrile (CH_3_CN, ACS reagent, ≥99.5%), *N*,*N*-dimethylformamide (DMF, anhydrous, 99.8%), ethanol (C_2_H_5_OH anhydrous ≥99.5%), *N*-methyl pyrrolidinone (NMP, Biosynthesis OmniSolv®), polyvinyl fluoride (PVDF), chloroform (CHCl_3_, ACS reagent, ≥99.8%), lithium hexafluorophosphate (LiPF_6_), Li sheet, acetone (ACS reagent, ≥99.5%), dibutyl phthalate (DBP, 99%), ethylene carbonate (EC, battery grade, ≥99%, acid <10 ppm, H_2_O < 10 ppm) and dimethyl carbonate (DMC, battery grade, ≥99.9%, acid <10 ppm, H_2_O < 10 ppm) were purchased from Sigma-Aldrich company. Outer diameter and length (O.D. x L) of MWCNT and MWCNT-COOH were 6–13 nm × 2.5–20 µm, and 9.5 nm × 1.5 µm, respectively.

### 2.1 Synthesis of the PDPA/MWCNT-COOH-1 composite

**Fig. 1:**
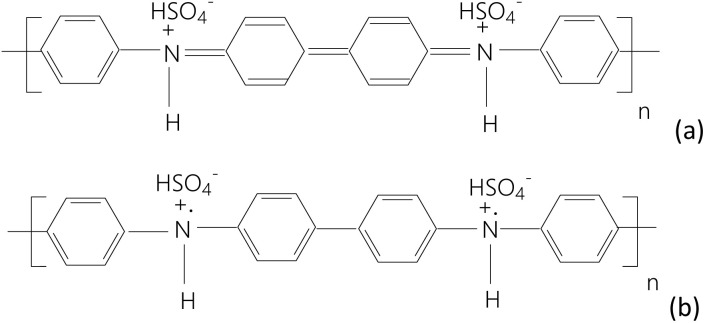
Chemical structure of PDPA-ES with repeating units containing quinodiimine with bipolaronic-type structure (a) and a *p*-diphenylenediamine with polaronic-type structure (b), both repeating units being doped with HSO_4_^−^ ions.

The synthesis method of the PDPA/MWCNT-COOH-1 composite is similar to that of PDPA, through the chemical polymerization of DPA in the presence of multi-walled carbon nanotubes (MWCNTs). The polydiphenylamine-emeraldine salt (PDPA-ES) synthesis method involves the dissolution of 1.12 mM DPA in a solution of 50 ml H_2_SO_4_ 0.5 M and 10 ml of DMF, resulting in a colorless solution, and 2.678 mM K_2_Cr_2_O_7_ in 50 ml H_2_SO_4_ 0.5 M, when an orange solution results. The two solutions are mixed and homogenized under ultrasound for 5 min, when the solution turns green. The reaction mixture is left to interact under magnetic stirring for 2 hours, when a dark green precipitate is observed, which is later filtered, washed with 500 ml H_2_O and 100 ml CH_3_CN, and dried to constant weight. Similar to the chemical mechanism of the oxidative polymerization of DPA in the presence of ammonium persulfate ((NH_4_)_2_S_2_O_8_) and H_2_SO_4_ (ref. 18) also occurs in the case of the chemical polymerization of DPA in the presence of K_2_Cr_2_O_7_ and H_2_SO_4_. Briefly, it involves the oxidation of DPA with the formation of a cation radical, a species that is unstable and which, through dimerization, can lead to both a quinodiimine with bipolaronic-type structure and a *p*-diphenylenediamine with polaronic-type structure. The growth of the macromolecular chain leads to the obtaining of such a PDPA-ES having structural units such as those shown in Fig. 1.

The synthesis of PDPA-EB involved the same steps as in the case of PDPA-ES, with the only difference that after the step of filtering the dark green precipitate corresponding to PDPA-ES, the interaction with 100 ml NH_4_OH 1 M takes place, the step which is then followed by washing with 500 ml H_2_O and 100 ml CH_3_CN, and then drying to constant mass. The solubility of PDPA in the organic solvent *N*-methyl pyrrolidinone (NMP) was equal to 3 mg ml^−1^ at a temperature of 25 °C.

In the case of PDPA/MWCNT-COOH-1 composite, the synthesis involves the addition of 0.04 g of MWNT in the solution of DPA in H_2_SO_4_ and DMF, the other steps taking place as above, the final product corresponding to the mixture of PDPA/MWCNT-COOH and MWCNT-COOH (labelled as PDPA/MWCNT-COOH-1 composite) as will be shown below. The preparation of PDPA/MWCNT-COOH-1 composite was repeated at least 3 times.

### 2.2 Preparation of electrodes and assembly of lithium rechargeable batteries

The electrodes were prepared by mixing 80 wt% active material, *i.e.* the PDPA/MWCNT-COOH-1 sample, with PVDF (5 wt%) and C Super P (15 wt%) in the presence of NMP, which was homogenized under magnetic stirring for 24 hours. The resulting solution was deposited on Al current collectors using the “Doctor blade” method to obtain films with a thickness of 180 µm. The electrode was dried at 60 °C for 4 hours. After drying, electrodes were obtained in the form of disks with a diameter of 10 mm. The discs were dried in a vacuum at 80 °C for 24 hours. The electrolyte used was LiPF_6_ in a 50 : 50 volumetric mixture of EC and DMC. The choice of an organic aprotic electrolyte (LiPF_6_ in EC : DMC 50 : 50) has been motivated by its wide electrochemical stability window, which enables operation at higher cell voltages and thus higher energy density compared to protic acidic electrolytes.^19,20^ Besides, the absence of protons minimizes parasitic reactions and ensures chemical compatibility with charge storage mechanisms in RLIBs. The cells were assembled by adding 80 µL of electrolyte in an Ar-filled glovebox from MBraun, with a diameter of 10 mm was used as anode material.

### 2.3 Synthesis of the PDPA/MWCNT-COOH-2 composite from commercial products

The synthesis of the PDPA/MWCNT-COOH composite from commercial products took place similarly to the aforementioned protocol. In the case of the PDPA/MWCNT-COOH-2 composite, the amount of MWCNT-COOH is equal to 0.02 g in the reaction mixture. The preparation of PDPA/MWCNT-COOH-2 composite was repeated at least 3 times.

### 2.4 The extraction of PDPA/MWCNT-COOH and MWCNT-COOH from EoL rechargeable lithium-ion battery (RLIB) cathodes

Considering the chemical composition of the spent RLIB cathode, consisting of PDPA/MWCNT-COOH-1 (*i.e.*, PDPA/MWCNT-COOH and MWCNT-COOH), PVDF, carbon Super P, and that during the charge–discharge processes, the chemical adsorption of LiPF_6_ and the formation of Li_2_CO_3_ and Li_2_O on the electrode surface took place,^21^ the following protocol was used for the separation of the constituents after the disassembly of the cell. After collecting 0.23 g of electrodes containing as active material the composite PDPA/MWCNT-COOH and MWCNT-COOH, a wash with 100 ml of distilled water took place in order to eliminate salts adsorbed onto the electrode surface, *i.e.*, LiPF_6_, Li_2_CO_3_, and Li_2_O. By ultrasonication of electrodes containing PDPA/MWCNT-COOH, PVDF, C Super P, and MWCNT-COOH in 50 ml NMP for 20 min. and then filtration, the separation of C Super P by the solution of MWCNT-COOH, PDPA/MWCNT-COOH, and PVDF took place. The solution of PDPA/MWCNT-COOH, MWCNT-COOH, and PVDF in NMP (50 ml) was interacted with C_2_H_5_OH (10 ml), known as a non-solvent for PVDF, and then, by filtration, PVDF was separated from the solution of PDPA/MWCNT-COOH and MWCNT-COOH in NMP : C_2_H_5_OH 5 : 1 (volumetric ratio). By a controlled evaporation of NMP and C_2_H_5_OH, a powder of the mixture of PDPA/MWCNT-COOH and MWCNT-COOH was obtained. This will be used to prepare new electrodes to be used in symmetric supercapacitor cells. The extraction protocol of the PDPA/MWCNT-COOH and MWNT-COOH composite from RLIB cathodes at EoL was repeated 2 times, after collecting 0.23 g of electrodes.

### 2.5 The assembly of supercapacitor cells

The performance testing of the electrodes prepared above was carried out using symmetrical supercapacitor cells, in which the two electrodes contain as active material the composite PDPA/MWCNT-COOH-1 or PDPA/MWCNT-COOH-2 and as electrolyte a Nafion membrane activated with H_2_SO_4_ 0.5 M according to the Savinell method.^22^ The choice of protic acid electrolyte, such as Nafion membrane activated with H_2_SO_4_, was made taking into account that such electrolytes are limited by proton-driven side reactions and a narrow voltage window, which makes them more suitable for electrochemical supercapacitor cells than for RLIBs.^23^ The potential range in which the cyclic voltammograms were recorded was (−0.2; +0.96) V. The electrodes were prepared according to the protocol published in ref. 24. This protocol for the preparation of the electrodes involved the use of 80% active material, 5% PVDF as a binding agent, 15% C Super P, 2 drops of DBP, and 1 ml of acetone. These compounds were mixed for 12 hours to obtain a homogeneous paste. The paste was spread on the surface of a glass slide, and after acetone evaporation, the resulting film was peeled and washed with diethyl ether to remove DBP, the film being cut into circles with a diameter of 8 cm. The electrode preparation was repeated at least 4 times. The electrodes were assembled in Swagelok cells. In order to eliminate the short circuit, a plastic sleeve was used as an insulator between the collector and the steel support of the Swagelok cells. A comparative evaluation of the performance of supercapacitors with PDPA/MWCNT-COOH-1 and PDPA/MWCNT-COOH-2 composites as active materials is presented using PDPA-ES and PDPA/MWCNT. The PDPA/MWCNT composite was obtained by solid-state interaction of PDPA-ES with MWCNT, by grinding, resulting in PDPA/MWCNT composites with a MWCNT concentration of 5 wt%.

### 2.6 Optical, structural, and electrochemical characterization of composite materials

The optical characterization of the PDPA/MWCNT-COOH-1 and PDPA/MWCNT-COOH-2 composites was performed by Raman scattering and FTIR spectroscopy. Raman spectra were recorded with the FT Raman spectrophotometer, MultiRaman model, from Bruker, at the excitation wavelength of 1064 nm. FTIR spectra were recorded with the FTIR spectrophotometer, model Vertex 80, from Bruker.

The structural properties were highlighted by X-ray diffraction (XRD). The XRD diagrams were obtained with an Anton-Paar XRDynamic 500 diffractometer, with Cu K(α) radiation (*λ* = 1.5406 Å), equipped with a 1D Pixos 2000-type detector. The measurements were recorded in a Bragg–Brentano monochromator configuration using a step of 0.03° and a dwell time of 1 s per pixel.

In the case of XPS spectra, a SPECS type XPS spectrometer was used, equipped with a PHOIBOS 150 analyzer and a 300 W monochromatic RX source, Al Kα – 1486.61 eV.

TG and DSC analyses were performed with a simultaneous thermal analysis system, STA 449 F3 Jupiter from Netzsch, in TG-DSC mode. Samples with masses of approx. 6–8 mg were introduced into alumina crucibles, uncovered and heated from room temperature to 1000 °C in an inert Argon atmosphere, using a gas flow rate of 20 ml min^−1^. For all samples, the standard heating rate of 10 °C min^−1^ was used. The accuracy of the heat flux measurements (DSC signal) was ±0.001 mW, and the temperature accuracy was ±0.01 °C. Samples were also investigated in synthetic air (20% O_2_ and 80% N_2_), from room temperature to 1000 °C, using a heating rate of 10 °C min^−1^ and a heat flow rate of 20 ml min^−1^.

Testing of rechargeable lithium batteries was performed by galvanostatic charge/discharge measurements using a NEWARE BTS4000 battery cycler.

The electrochemical supercapacitors were tested using cyclic voltammetry, the voltammograms being recorded with an Origaflex multi-channel potentiostat from Origalys.

To ensure reproducibility of results, the Raman and FTIR spectra were collected from at least three independent samples. In the case of Raman spectra, for each sample Raman spectra were recorded by 200 of accumulations, the laser power being equal to 25 mW. In the case of FTIR spectra, each spectrum was recorded using 64 accumulations. The XPS spectra, XRD, and TG-DSC diagrams were collected only by one run. In the case of the electrochemical measurements, these were repeated 2 times on independently prepared samples.

## 3. Results and discussion

### 3.1 Optical and structural characterization of PDPA/MWCNT-COOH-1 composites and their applications in the RLIBs field

**Fig. 2:**
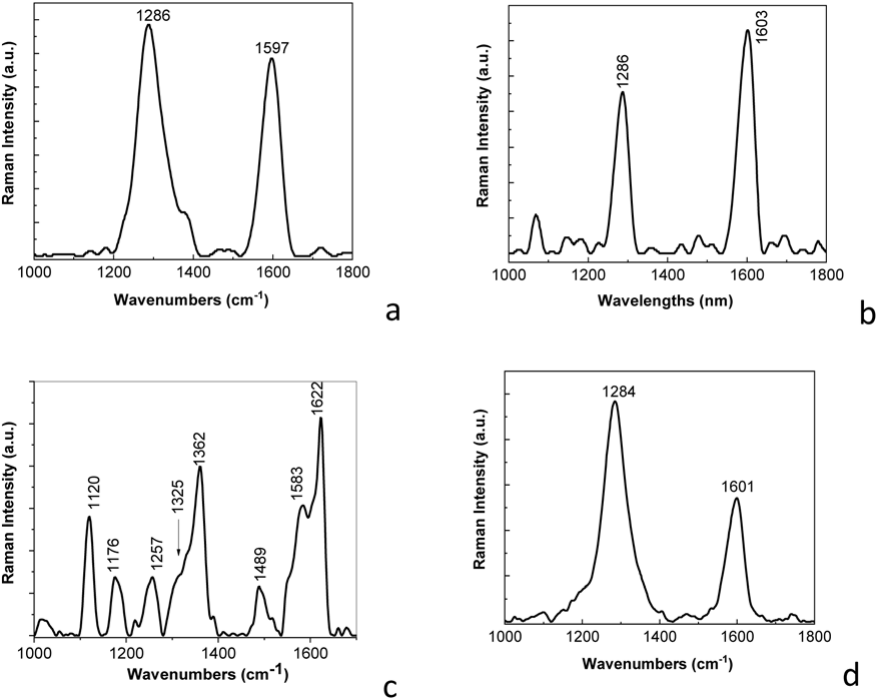
Raman spectra of MWCNT (a), the PDPA/MWCNT-COOH-1 composite (b), PDPA (c), and MWCNT-COOH (d).

Fig. 2 shows Raman spectra of the PDPA-ES, PDPA/MWCNT-COOH-1, and MWCNT. According to Fig. 2a, the Raman spectrum of MWNT highlights two Raman lines at 1286 and 1597 cm^−1^, assigned to the defects and/or disorder state in the graphitic lattice of MWCNT (labelled as D band) and the tangential vibration mode (labelled as G band), whose ratio between the intensities of the D and G bands is equal to 1.17.^25^

The D and G bands of the Raman spectrum of MWCNT-COOH are peaked at 1284 and 1601 cm^−1^ (Fig. 2d). The ratio between the D and G bands in the case of MWCNT-COOH is equal to 1.79. The increase in the intensity of the D band is a consequence of the defects induced by the presence of carboxylic groups on the MWCNT surface, when some C=C bonds involving carbon atoms with sp^2^ hybridization are transformed into C–C bonds involving carbon atoms with sp^3^ hybridization. Raman spectrum of the PDPA/MWCNT-COOH-1 composite (Fig. 2b) highlights that the Raman line related to the tangential vibrational mode of MWCNT is shifted to 1603 cm^−1^. An explanation for this behavior must take into account that PDPA shows a Raman line at 1610 cm^−1^ assigned to the vibrational mode C–C stretching in the benzene (B) ring.^24^ A first argument that the surface of MWCNTs is co-functionalized with carboxylic groups and PDPA consists in the change in the ratio between the intensities of the Raman lines from 1286 and 1603 cm^−1^, this being subunitary (0.72) in the case of the PDPA/MWCNT-COOH-1 composite, unlike the supra-unitary values of this ratio in the case of MWCNT (Fig. 2a) and MWCNT-COOH (Fig. 2d). This change can be explained considering: (a) a potential interaction of carboxylic groups of MWCNT-COOH with amine groups of PDPA; (b) coating MWCNT-COOH with PDPA, when there is a reduction in Raman light scattering from areas related to the defect state of carbon nanotubes; in this context, we observe in the Raman spectrum of PDPA-ES (Fig. 2c), that the most intense line is peaked at 1622 cm^−1^, this being assigned to the vibrational mode C–C stretching in the benzene ring.^26^ The other Raman lines of PDPA-ES having maxima at 1120, 1176, 1257, 1325, 1362, 1489 and 1583 cm^−1^ have been assigned to the vibrational modes C–C stretching + C–H bending, C–H bending in the benzene ring, C_aromatic_–N in the entities of the type *N*,*N*′-diphenylbenzidine radical cations, C–C stretching (B) + C–H bending (B), C=N stretching, and C=C stretching in the quinoid ring;^26,27^ and (c) π–π interactions at the MWNT-COOH and PDPA interface that can partially restore the sp^2^ conjugation.

**Fig. 3:**
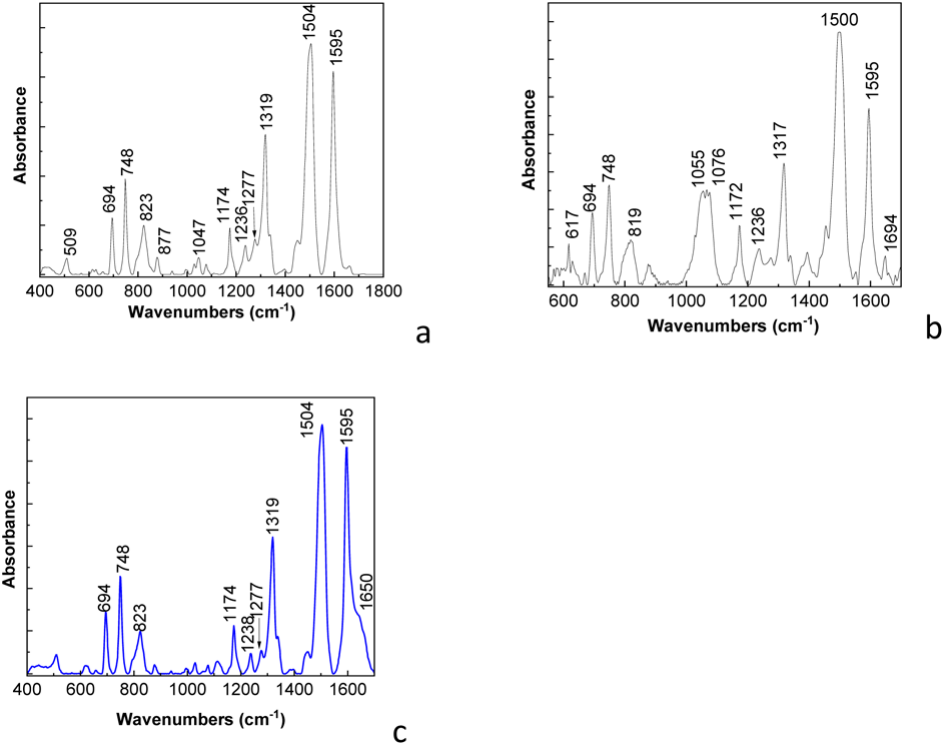
FTIR spectra of PDPA-ES (a), PDPA/MWCNT-COOH-1 (b) and PDPA-EB (c).

Complementary information regarding the vibrational properties of the PDPA/CNT composite is further presented by FTIR spectroscopy in Fig. 3.

Fig. 3a shows the FTIR spectrum of PDPA-ES, which highlights IR bands with maxima at 694–748, 823, 1174, 1236, 1277, 1319, 1504, and 1595 cm^−1^, that are assigned to the vibrational modes of the monosubstituted phenyl ring, the out-of-plane C–H bending vibration of 4, 4′-substituted ring, in-plane bending C–H of aromatic ring, C_aromatic ring_–N bond, stretching C–C between two rings, C=N bond, C–C stretching in 4,4′-substituted benzoid ring and quinoid ring, respectively.^18,28,29^ The IR band, which peaked at 1047 cm^−1^, is assigned to the vibrational mode of the S=O bond.^18^Fig. 3b shows the FTIR spectrum of the PDPA/MWCNT-COOH-1 composite, which highlights IR bands at ∼694–748, 819, 1055, 1076, 1172, 1317, 1500, 1595, and 1694 cm^−1^. The shift of the IR band from 1047 cm^−1^ to 1055 cm^−1^ is induced by the presence of an IR band from 1076 cm^−1^, which is not far from the one reported at 1095 cm^−1^, which has been assigned to the vibrational mode of the C=C bond in MWCNT.^30^ The IR band from 1694 cm^−1^ is assigned to the carboxylic groups (-COOH), which appear on the MWCNTs surface as a consequence of the interaction of MWCNT with K_2_Cr_2_O_7_ and H_2_SO_4_.^31^ A first conclusion of these studies is that the chemical polymerization of DPA in the presence of MWCNT leads to a mixture of MWCNT-COOH and MWCNT-COOH functionalized with PDPA (PDPA/MWCNT-COOH). According to Fig. 3c, the FTIR spectrum of PDPA-EB shows the main differences compared to the FTIR spectrum of PDPA-ES, the absence of the IR band at 1047 cm^−1^, and the presence of the IR band at 1650 cm^−1^, assigned to quinodiimine groups.^18^ Using to calculate the degree of oxidation, the formula used in the case of polyaniline.^32^*x* + [*x*(*A*_Q_/*A*_B_)] = 1,where *x*, *A*_Q_, and *A*_B_ correspond to the degree of oxidation, the absorbance of the IR bands at 1595 and 1504 cm^−1^ attributed to vibrational modes C–C stretching deformation in 4,4′-substituted quinoid and benzoid ring, we determined that the degree of oxidation is 0.524.

**Fig. 4:**
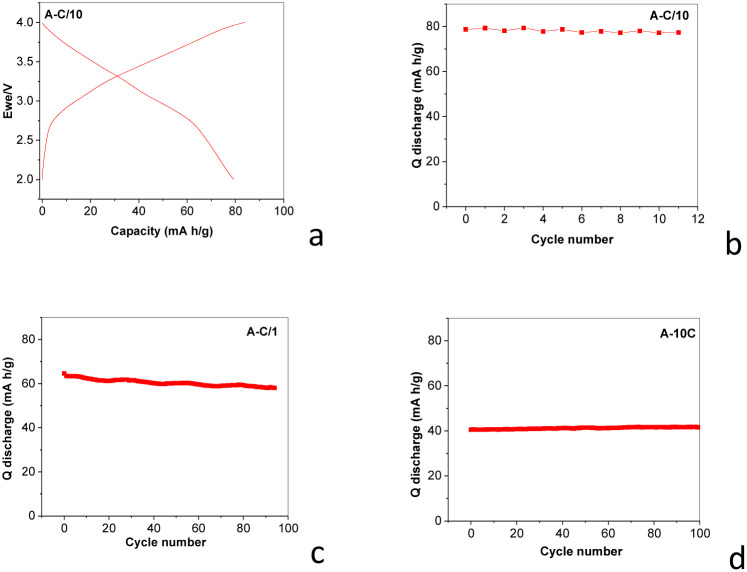
Galvanostatic charge–discharge curves of RLIB of the type (−)Li/PDPA/MWCNT-COOH-1(+) composite (a). The evolution of the discharge capacity as a function of the cycles number at 0.1C (b), 1C (c), and 10C (d).

As noted above, this mixture will be labelled further as the PDPA/MWCNT-COOH-1 composite. The testing of the PDPA/MWCNT-COOH-1 composite as an active material in the cathode of RLIB until the stage of the EoL is presented in Fig. 4.

Thus, Fig. 3 shows the galvanostatic charge–discharge curves of a lithium-ion cell of the type (−)Li/PDPA/MWCNT-COOH-1(+) composite, obtained at a constant current density. The characteristic shape of the curves highlights the reversible redox processes associated with the electrochemical cycling of the mixture of MWCNT-COOH and PDPA/MWCNT-COOH-1 composite. Fig. 4 shows the presence of well-defined voltage plateaus both during the charging and discharging processes, which suggests the existence of intercalation and de-intercalation processes of Li^+^ ions relatively well defined energetically. The stability and reproducibility of these plateaus over successive cycles indicate a good reversibility of the redox reactions, specific to the interaction between the conductive polymer matrix and the carbon phase. The potential differences between the charge and discharge branches (electrochemical hysteresis) are moderate, denoting an acceptable internal resistance and relatively low energy losses. However, a gradual increase in polarization at subsequent cycles could indicate the progressive formation of the solid–electrolyte interfacial layer or a slight degradation of the electrical contact between the active phases. The comparative analysis of Fig. 4b–d shows that the general shape of the curves is preserved, but a slight decrease in the discharged capacity can be observed, a phenomenon attributed to possible initial irreversible processes and microstructural restructuring of the composite cathode. This trend is typical of organic and hybrid materials, where long-term stability depends on the balance between polymer flexibility and carbon network stiffness. The discharge capacity varies from 80 mA h g^−1^ to 40 mA h g^−1^ when the load increases during the charging/discharging processes.

### 3.2 Optical and structural characterization of the compounds separated from the cathodes of the spent RLIB

To highlight the chemical compounds recovered from the RLIB cathodes that reached after 100 galvanostatic charge–discharge (GCD) cycles at capacitance values of 40 mA h g^−1^, the Raman spectra of the recovered compounds are presented below.

**Fig. 5:**
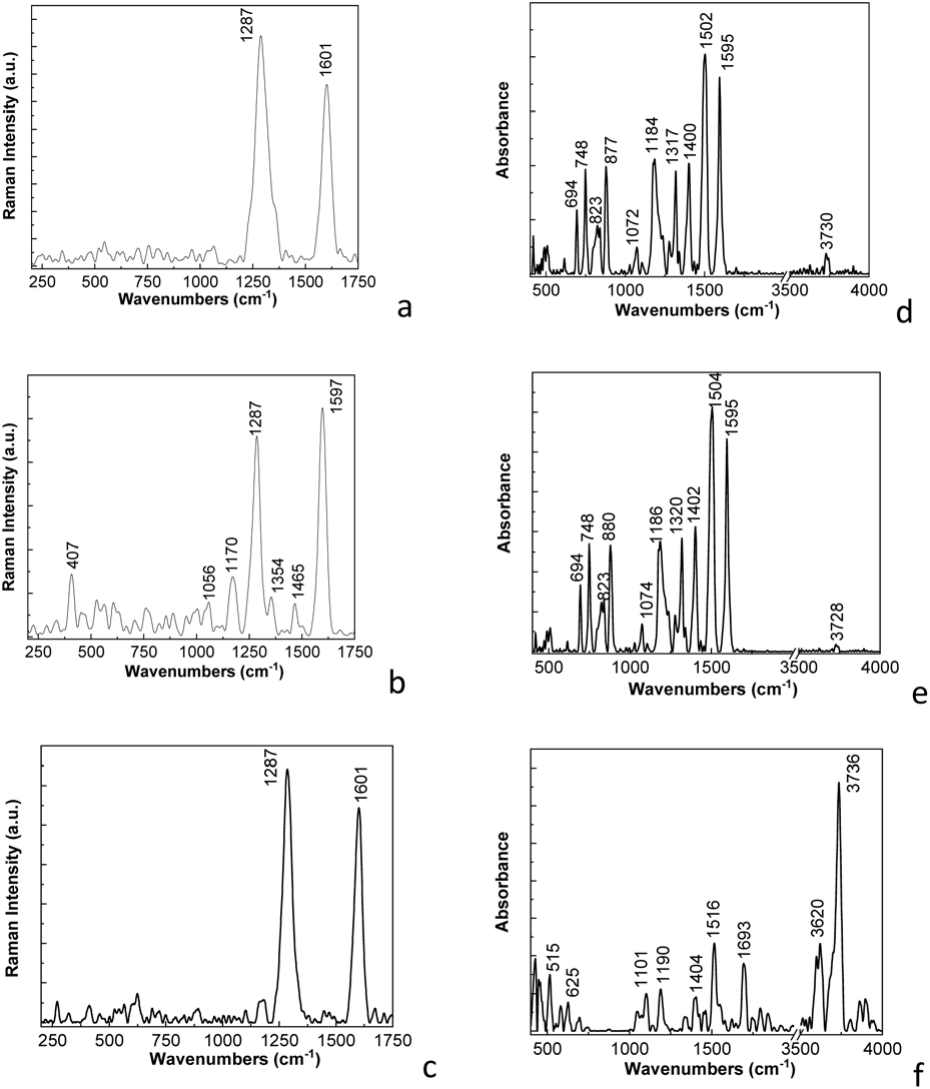
Raman spectra of: (a) the electrode containing PDPA/MWCNT-COOH-1 (PDPA/MWCNT-COOH, and MWCNT-COOH), PVDF, carbon Super P, Li_2_CO_3_, Li_2_O, and LiPF_6_; (b) PDPA/MWCNT-COOH-1, and PVDF; and (c) PDPA/MWCNT-COOH-1, and MWCNT-COOH, labelled as PDPA/MWCNT-COOH-2. FTIR spectra of: (d) the electrode containing PDPA/MWCNT-COOH-1 (PDPA/MWCNT-COOH, and MWCNT-COOH), PVDF, carbon Super P, Li_2_CO_3_, Li_2_O, and LiPF_6_; (e) PDPA/MWCNT-COOH-1, and PVDF; and (f) PDPA/MWCNT-COOH-1, and MWCNT-COOH, labelled as PDPA/MWCNT-COOH-2.

Fig. 5 shows the Raman and FTIR spectra of the compounds recovered from the cathodes of RLIB at EoL.

Thus, Fig. 5a shows the Raman spectrum of the cathode after the elimination of inorganic compounds such as LiPF_6_, Li_2_CO_3,_ and Li_2_O. According to Fig. 4a, the Raman spectrum is dominated by two intense bands with maxima located at 1287 and 1601 cm^−1^, which correspond to the vibration modes of carbon atoms with sp^3^ hybridization (labelled as D band) and the respective sp^2^ (labelled as G band) existing in the compounds of the type C Super P, and PDPA/MWCNT-COOH-1.^25,33^ According to Fig. 5a, the ratio between the intensities of the D and G bands is equal to ∼1.27. Fig. 5b shows the SERS spectrum of the mixture of PVDF and PDPA/MWCNT-COOH-1 deposited as a film of approx. 200 nm, on Au support, prepared according to ref. 34, by evaporating NMP under high vacuum for 2 hours. Fig. 5b illustrates the presence of the following Raman lines located at:

(i) 407, 1170, 1354, and 1465 cm^−1^, which belong to PDPA being attributed to the vibrational modes of deformation of the aromatic ring of the polymer, the vibration of the C–H bond in the benzene ring (A_g_ model), the protonated structure of the polymer, and the stretching vibration of the C=C bond in the quinoid ring of PDPA;^21^

(ii) 1287 and 1597 cm^−1^, which correspond to the D and G bands of carbon nanotubes in PDPA/MWCNT-COOH-1 composite; the ratio between the intensities of bands D and G becomes equal to 0.94 (Fig. 5b), a value which is lower than that determined in the case of Fig. 5a, as a consequence of the elimination of C Super P, with the amorphous structure, which shows an important contribution to the intensity of the D band, allowing the highlighting of the defect states of the MWCNTs co-functionalized with carboxyl groups and PDPA; and

(iii) 1056 cm^−1^, which belongs to the vibration mode of symmetric stretching of the CF_2_ bond and swinging of the CH_2_ bond of PVDF.^35^

Fig. 5c shows the Raman spectrum of PDPA/MWCNT-COOH-2, which is characterized by the two Raman lines at 1287 and 1601 cm^−1^, having a ratio of the two bands equal to ∼1.2, a value that is higher than that mentioned in the case of the PDPA/MWCNT-COOH-1 composite prepared from commercial compounds (0.72, Fig. 2b). This result indicates a higher disorder in the case of the PDPA/MWCNT-COOH-2 composite recovered from the RLIB cathode than that prepared from commercial compounds, *i.e.*, PDPA/MWCNT-COOH-1, which, in our opinion, can be explained by considering both the processes that occur during the GCD cycles and during the separation of the composite from the RLIB cathode. In this context, we believe that a contribution to the higher disorder state of the PDPA/MWCNT-COOH-2 composite can be given by: (i) the mechanical stress induced during the GCD cycles, when defects in the MWCNT walls are produced; (ii) the interaction of the composite with Li_2_CO_3_ which is generated during GCD cycles and can lead to the appearance of new C–C bonds having the carbon with sp^3^ hybridization on the MWCNT surface; (iii) the insertion/de-insertion of Li^+^ ions which distort the sp^2^ lattice of the MWCNT, and last but not least; (iv) the ultrasonication processes used during the separation steps of the composite from the other constituents of the cathode.

Fig. 5d shows the IR spectrum of the cathode containing PDPA/MWCNT-COOH-2, PVDF, carbon Super P, Li_2_CO_3_, Li_2_O, and LiPF_6_, which highlights the following IR bands with maxima located at 694, 748, 823, 877, 1072, 1184, 1317, 1400, 1502, 1595, and 3730 cm^−1^. The IR bands at 877, 1070, 1184, and 1400 cm^−1^ (Fig. 5d) are located in the vicinity of the IR bands of PVDF, which have maxima at ∼877, 1070, 1186 and 1404 cm^−1^, they being attributed to the vibrational modes of asymmetric stretching of the C–C and CF_2_ bonds, the symmetric stretching vibration of the C–C bond + the rocking vibration of the bonds – CF_2_ and CH_2_, and respectively the rocking vibration of the CH_2_ bond and the asymmetric stretching of the C–C bond specific to the β crystalline form and/or γ of PVDF.^36,37^ The IR band at 3730 cm^−1^ in Fig. 5d is located near the IR band observed in the case of the mixture of PDPA/MWCNT-COOH-1, and PVDF (3728 cm^−1^, Fig. 5e) and of the PDPA/MWCNT-COOH-2 composite (−3736 cm^−1^, Fig. 5f). The IR bands at 694, 748, 1317, 1502 and 1595 cm^−1^ (Fig. 5d) are located in the vicinity of those observed in Fig. 3 which are located at 694, 748, 1320, 1504 and 1595 and 1595 cm^−1^, which are characteristic of PDPA vibrations, these being attributed to the vibrational mode of inter-ring deformation, the deformation of the benzene ring, the stretching of the C_aromatic_–N bond, the stretching of the C–C bond + the C–H bond and the stretching of the C–C bond in the benzene ring.^27,28^ The presence of the IR band from 1693 cm^−1^ in the PDPA/MWCNT-COOH-2 composite (Fig. 5f), having the absorbance superior to the IR band of the PDPA/MWCNT-COOH-1 composite (Fig. 3b), clearly proves the presence of a higher proportion of carboxyl groups in the case of the composite recovered from the spent RLIB cathode. The IR band at 3728–3730 cm^−1^ is assigned to the O–H stretching vibrational mode of the carboxylic groups in PDPA/MWCNT-COOH-1. Its absorbance is very low as a consequence of the presence in the vicinity of the PDPA/MWCNT-COOH-1 composite of other compounds of the type: (i) PVDF, and C-Super P, when processes of shielding of O–H sites from composite by PVDF and C-Super P can occur and (ii) the formation of new hydrogen bonds between the OH bond of the PDPA/MWCNT-COOH-1 composite and the oxygen of Li_2_O can take place. In the case of the FTIR spectrum of PDPA/MWCNT-COOH-2 (Fig. 5f) one observes that the IR band assigned to the O–H stretching vibrational mode of the carboxylic groups shifts to 3736 cm^−1^, simultaneously with its increase in absorbance and the appearance of a new IR band at 3620 cm^−1^. These changes indicate the absence of the shielding process of the O–H sites from the PDPA/MWCNT-COOH-2 composite, facilitating the formation of intermolecular hydrogen bonds, of the type H⋯N–H and N–H⋯O.

**Fig. 6:**
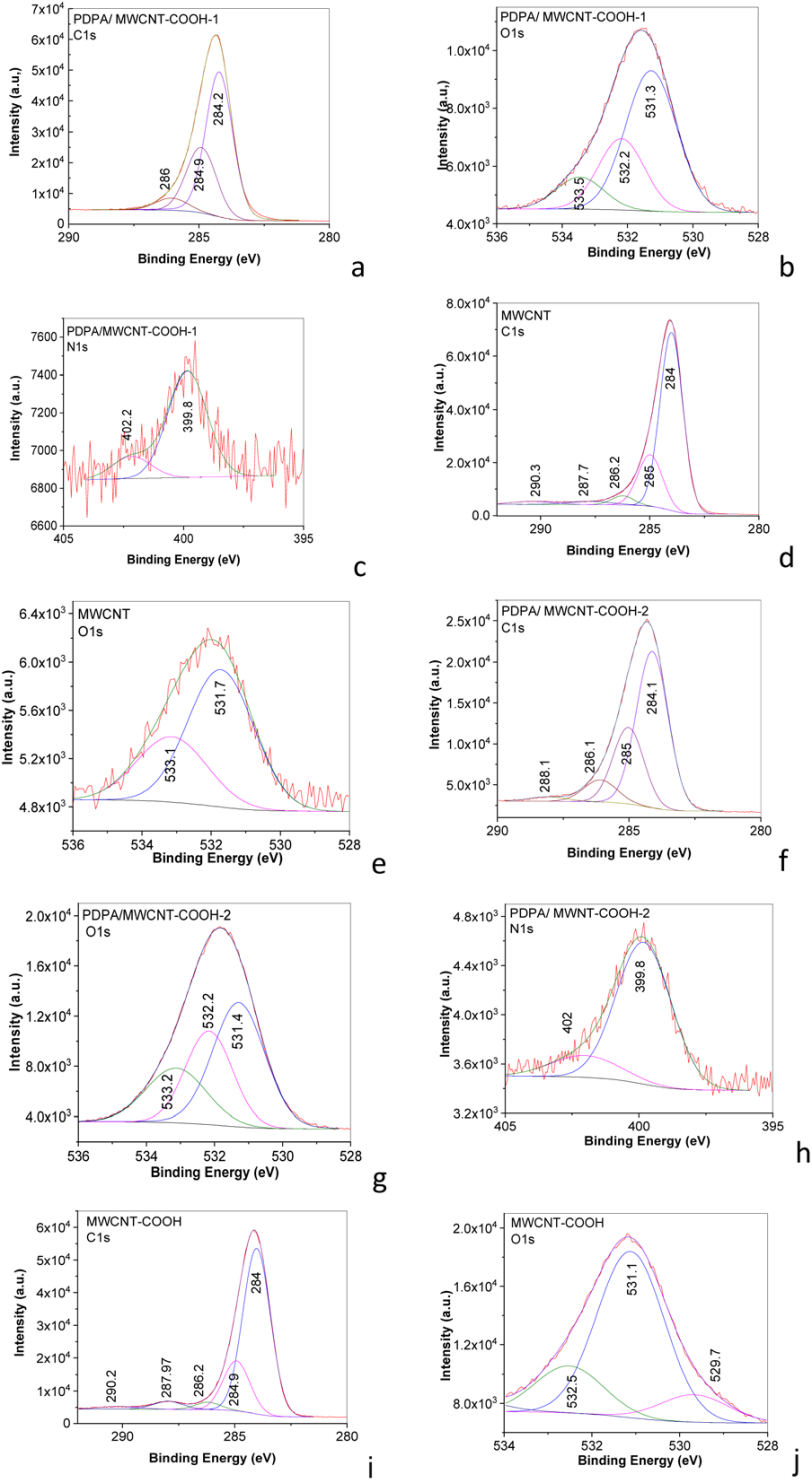
XPS C 1s, O 1s, and N 1s spectra of the mixture of PDPA/MWCNT-COOH-1 and MWCNT-COOH (a–c), MWCNT (d and e), PDPA/MWCNT-COOH-2 (f–h), and MWCNT-COOH (i and j).

The additional information concerning the bonds existing in MWCNT, MWCNT-COOH, PDPA/MWCNT-COOH-1, and PDPA/MWCNT-COOH-2 is shown by XPS in Fig. 6.

**Fig. 7:**
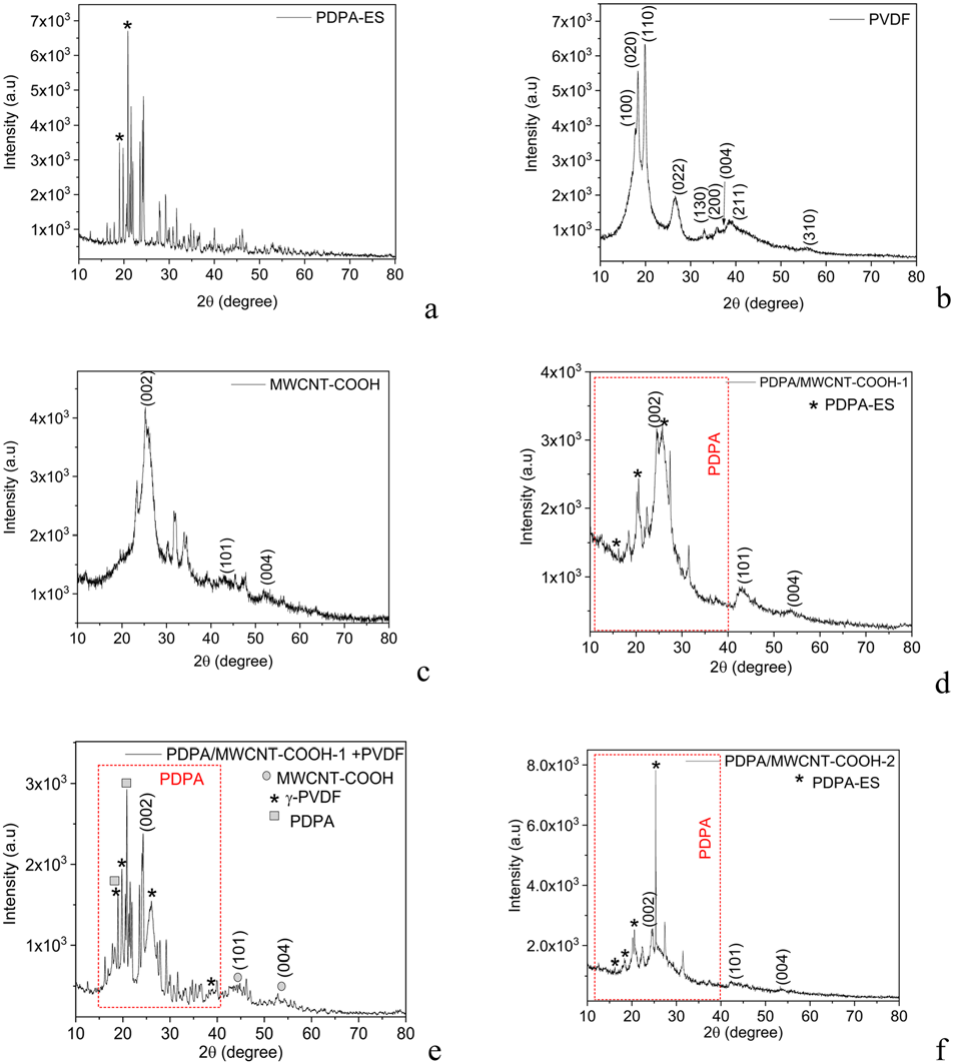
XRD diagrams of: (a) PDPA-ES, (b) PVDF; (c) MWCNT-COOH; (d) the PDPA/MWCNT-COOH-1 composite, (e) the sample containing PDPA/MWCNT-COOH-1 and PVDF; and (f) PDPA/MWCNT-COOH-2.

The deconvoluted XPS C 1s spectra of MWCNT and MWCNT-COOH show five peaks at 284, 285, 286.2, 287.7–287.9, and 290.3–290.2 eV, which are assigned to the sp^2^ hybridized carbon, sp^3^ hybridized carbon in C–C bond, C–N bonds, C–O/C=O bonds, and π–π transitions.^38,39^ The deconvoluted XPS O 1s spectrum of MWCNT highlights two peaks at 533.1 and 531.7 eV, assigned to the C–O bond/adsorbed water molecules and C=O bond,^38^ while in the case of MWCNT-COOH, XPS O 1s spectrum shows three peaks at 532.5, 531.1, and 529.7 eV belonging to the bonds C–O and C=O in the carboxylic group and C=O in quinone state.^38,40,41^ Detailed analysis of the XPS spectra of the two composites shows that:

(a) The deconvolution of the XPS C1s spectra of the PDPA/MWCNT-COOH-1 composite is characterized by three peaks located at 284.2, 284.9, and 286 eV, while in the case of the PDPA/MWCNT-COOH-2 composite, the peaks are at 284.1, 285, 286.1, and 288.1 eV. The peaks at 284.2–284.1, 284.9–285, 286.1–286, and 288.1 eV are assigned to the bonds C=C, C–C/C–H, C–O/C–N, and C=C–O/COOH.^41^

(b) The deconvolution of the XPS O1s spectra highlights, in the case of the composites: (b_1_) PDPA/MWCNT-COOH-1, three peaks located at 531.3, 532.2, and 533.5 eV, and (b_2_) PDPA/MWCNT-COOH-2, three peaks located at 531.4 eV, 532.2 eV, and 533.2 eV. The three peaks situated at 531.4–531.3, 532.2, and 533.5–533.2 eV have been assigned to the bonds C=O, C–O–C, and C–O– in COOH.^38^

(c) The deconvolution of the XPS N 1s spectrum of the PDPA/MWCNT-COOH-1 composite highlights two peaks at 399.8 eV and 402.2 eV, which, in the case of the PDPA/MWCNT-COOH-2 composite, are located at 399.8 eV and 402 eV. The two peaks at 402 and 399.8 eV are attributed to the C–N^+^ and C–N bonds.^42^ The ratio between the area of the peaks at 402 and 399.8 eV in the case of the composites PDPA/MWCNT-COOH-1 and PDPA/MWCNT-COOH-2, is equal to 0.46 and 0.72. This result indicates that a pseudo-protonic doping process takes place in the case of the composite recovered from the spent RLIB, which must be understood as an interaction between the PDPA/MWCNT-COOH-1 composite with MWCNT-COOH. The influence of these modifications reported by XPS studies will be correlated in the following with the information resulting from XRD analyses. Thus, information about the crystalline structure of the composites PDPA/MWCNT-COOH-1 and PDPA/MWCNT-COOH-2 is shown in Fig. 7, which shows XRD diagrams of: (i) PDPA, (ii) PVDF, (iii) MWCNT-COOH, (iv) the PDPA/MWCNT-COOH-1 composite, (v) the mixture PDPA/MWCNT-COOH-1 and PVDF, and (vi) the PDPA/MWCNT-COOH-2 composite. The XRD spectra of the samples containing PDPA and PVDF show a crystalline structure.

The main peaks observed in XRD diagrams prove that: (i) PVDF is in the γ state [PDF 00-061-1406]; (ii) the most intense peak in the XRD diagram in the case of the MWCNT-COOH sample at 25.3° corresponds to the crystal plane (002), this being accompanied of other two peaks of low intensity at 2*θ* equal to 42.3° and 53.5° which have been assigned to the crystalline planes (101) and (104);^43^ and (iii) the peaks located at 18° and 21° correspond to the crystal planes (110) and (018) of PDPA.^44^ The peaks in the XRD diagrams characteristic of the (002) plane of MWCNT-COOH become of reduced intensity in the case of the PDPA/MWCNT-COOH-2 composite as a consequence of a diminished graphitic order, while the reflections associated with the crystalline phases of PDPA (110) and (018) are maintained, confirming the stability of the doped phase of the PDPA/MWCNT-COOH-1 composite. Based on the XPS and XRD studies, we conclude that the RLIB cycling process and the separation process of the composite from the used RLIB cathode introduced sp^3^ defects and modified the interactions between MWCNT–COOH and the PDPA/MWCNT-COOH-1 composite, without completely destroying the crystalline structure of the conducting polymer.

**Fig. 8:**
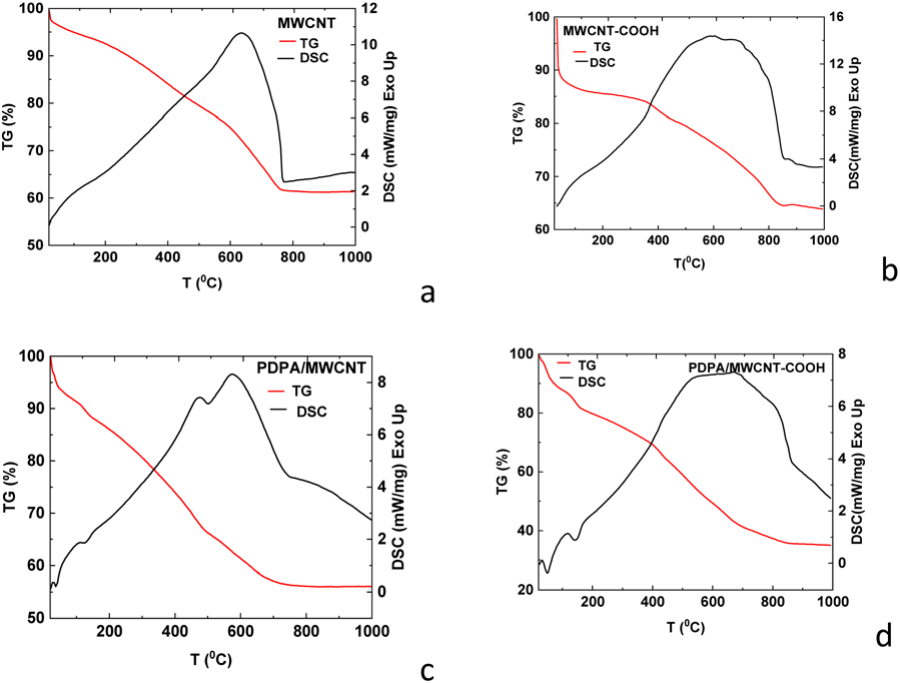
TG-DSC curves of the samples of MWCNT (a), MWCNT-COOH (b), PDPA/MWCNT-COOH-1 (c), and PDPA/MWCNT-COOH-2 (d) recorded in an inert Argon atmosphere.

Concerning the thermal stability of the two composites, Fig. 8 shows the TG-DSC curves, recorded in an inert Argon atmosphere, measured on the PDPA/MWCNT-COOH-1 and PDPA/MWCNT-COOH-2 composites, as well as MWCNT, and MWCNT-COOH.

Studies on the thermal stability of carbon nanotubes have highlighted some essential parameters that influence the thermal behavior of these materials, such as the working atmosphere (oxidative or inert), the heating rate, as well as the shape and thickness of the materials.^45^ According to the reported data, the thermal degradation of carbon nanotubes in an oxidizing atmosphere occurs at lower temperatures (*i.e.*, 500–700 °C) compared to measurements taken in an inert atmosphere, where the materials could be stable up to 800 °C. In our case, the two composites, PDPA/MWCNT-COOH-1 (Fig. 8c) and PDPA/MWCNT-COOH-2 (Fig. 8d), exhibited similar thermal behavior, in which the stability of the nanotubes is not significantly influenced by the groups with which they were functionalized. According to the characteristic TG-DSC curves, both samples exhibit a single-step thermal decomposition process, with mass losses of 44% for PDPA/MWCNT-COOH-1 and 65% for PDPA/MWCNT-COOH-2, respectively. The maximum decomposition temperatures are approximately 842 °C for the PDPA/MWCNT-COOH-1 sample and approximately 700 °C for PDPA/MWCNT-COOH-2. These values do not differ significantly from the MWNT (Fig. 8a) and MWCNT-COOH-2 (Fig. 8b) samples, respectively. These two samples are characterized by very close weight losses (38% MWCNT *versus* 37% MWCNT-COOH-2) and a difference of approximately 100 °C in decomposition temperatures, with a maximum of 755 °C for MWCNT *versus* 833 °C for MWCNT-COOH.

These results are consistent with the higher share of C–O–C bonds in the case of the PDPA/MWCNT-COOH-2 sample (Fig. 6g) in contrast with the PDPA/MWCNT-COOH-1 composite (Fig. 6b) and MWCNT-COOH (Fig. 6h), as observed in the XPS O1s spectra through the peak at 532.5 eV.

**Fig. 9:**
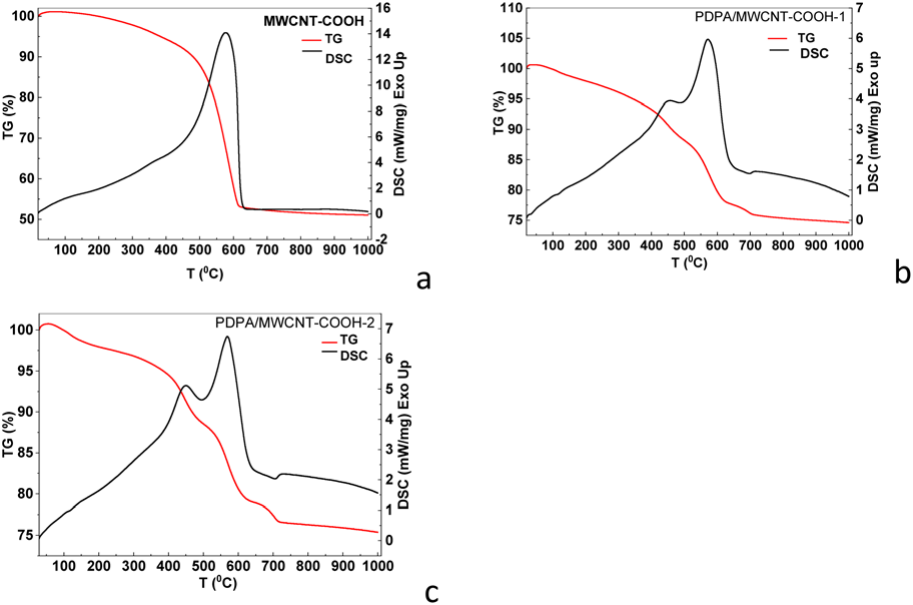
TG-DSC curves of the samples of MWCNT-COOH (a), PDPA/MWCNT-COOH-1 (b), and PDPA/MWCNT-COOH-2 (c).

Fig. 9 shows the TG-DSC curves in synthetic air (20% O_2_ and 80% N_2_).

According to Fig. 9b and c, the thermal behavior of PDPA/MWNT-COOH-1 and PDPA/MWCNT-COOH-2 is similar. The TG signals showed that both samples have very close residual masses (*e.g.*, 74.61% for PDPA/MWCNT-COOH-1 *vs.* 75.36% for PDPA/MWCNT-COOH-2), which means that there are no differences in their degradation mechanisms. This aspect is also confirmed by the two DSC curves, where it can be observed the presence of two exothermic peaks with very close values of their maxima (457 °C and 571 °C for PDPA/MWCNT-COOH-1 and 450 °C and 568 °C for sample PDPA/MWCNT-COOH-2), along with two endothermic peaks occurring at 698 °C for sample PDPA/MWCNT-COOH-1 and 705 °C for the second one. The MWNT-COOH sample (Fig. 9a) exhibits a one-step thermal degradation with a mass loss of ∼49% up to 630 °C accompanied by a pronounced exothermal peak on the DSC curve with the maximum around 577 °C. This behavior is similar to that observed in the Ar experiment, the only difference being that the thermal degradation temperature is lower in synthetic air compared to the inert atmosphere (*i.e.*, 577 °C *versus* 833 °C). Compared to the measurements carried out in an inert atmosphere, a decrease in the thermal stability of the materials can be observed, the thermal degradation in air being realized at lower temperatures (about 100 °C lower) due to oxidation processes that favor strongly exothermic reactions. These processes are confirmed by the behavior of the DSC curves, showing pronounced exothermic peaks in the reaction with synthetic air than in Ar. The TG curves showed lower mass losses for samples measured in the oxidative atmosphere than in Ar, due to the fact that, before thermal degradation starts, oxygen can interact with the surfaces of the materials, leading to the formation of oxygen-containing groups (*e.g.*, –C=O, –COO–, *etc.*) that can temporarily increase the masses.^46^

### 3.3 Preliminary studies on the use of composites PDPA/MWCNT-COOH as active electrode materials in supercapacitor cells

**Fig. 10:**
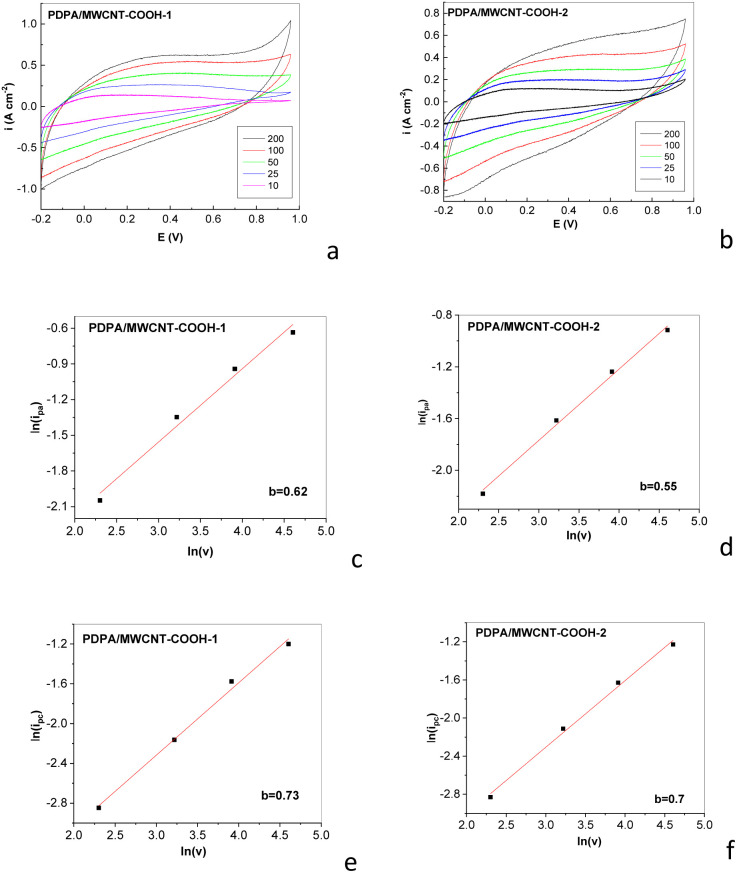
Dependence of cyclic voltammograms of symmetrical electrochemical supercapacitors having electrodes containing the PDPA/MWCNT-COOH-1 (a) and PDPA/MWCNT-COOH-2 (b) composites as active electrode material, when the scanning rate of the potential in the potential range (−0.2; +0.96) V is equal to 10, 25, 50, 100, and 200 mV s^−1^. Dependence of the anodic and cathodic currents on the scanning rate of potential in the case of the composites PDPA/MWCNT-COOH-1 (c and d) and PDPA/MWCNT-COOH-2 (e and f). In (c) and (e), the anodic current densities are read at a potential of +0.37 V, while in (d) and (f), the cathodic current densities are read at the potential of −0.37 V.

Fig. 10a and b highlight the dependence of the cyclic voltammograms on the scanning rate of the potential in the case of supercapacitors having electrodes containing the PDPA/MWCNT-COOH-1 and PDPA/MWCNT-COOH-2 composites as active material.

**Fig. 11:**
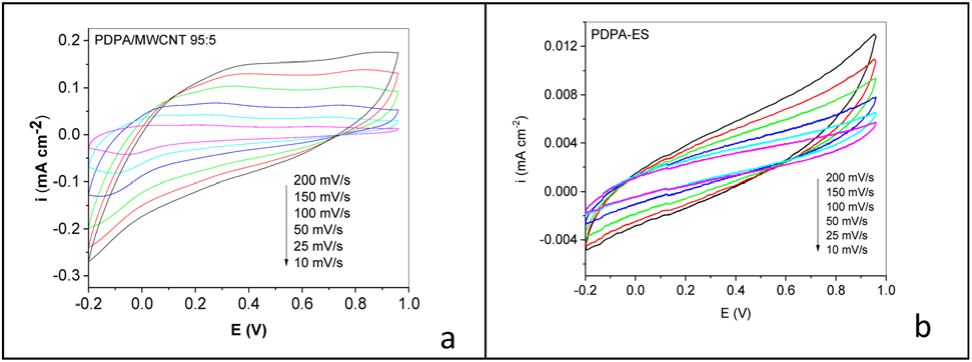
Dependence of cyclic voltammograms of symmetrical electrochemical supercapacitors having electrodes containing the PDPA/MWCNT composite (a) and PDPA-ES (b) as active electrode material, when the scanning rate of the potential in the potential range (−0.2; +0.96) V is equal to 10, 25, 50, 100, and 200 mV s^−1^.

Regardless of the active material of the electrodes, a rectangular profile of the cyclic voltammograms is observed in the two cases (Fig. 10a and b), which is characteristic of the electric double layer in the absence of a faradaic process. This rectangular profile of the cyclic voltammograms is also observed in the case of supercapacitors having the PDPA/MWCNT composite and PDPA-ES as electrode active material (Fig. 11a and b).

Fig. 10c and d highlight that in the cases of the PDPA/MWCNT-COOH-1 and PDPA/MWCNT-COOH-2 composites, a dependence of the anodic and cathodic current densities as a function of the scan rate of the potential is remarked. Generally, current densities show two components, one is related to the double-layer charge at the interface electrode/electrolyte interface, and another is induced by the intercalation processes, which are controlled by diffusion. The contribution of the two processes, *i.e.* capacitive and diffusion, can be determined using the equation: *i*(*V*) = *axv*^*b*^, where *i* and *v* correspond to current density (A cm^−2^) and potential scan rate (mV s^−1^), while *a* and *b* are two constants.^47^ The plot of ln(*i*) *vs.* ln(*v*) allows the determination of the values of the constants a and *b*. The value of the constant *b* can vary as follows: (a) when *b* = 1, which indicates that redox reactions occur at the electrode/electrolyte interface and that the charging/discharging processes of the electric double layer capacitors (EDLCs), (b) when 0.8 < *b* < 1, the active electrode material exhibits pseudocapacitive behavior, and (c) when 0.5 < *b* < 0.8, the active electrode material exhibits battery-like behavior.^37^ According to Fig. 10c–f, the electrodes based on composites PDPA/MWCNT-COOH-1 and PDPA/MWCNT-COOH-2 show a battery-like behavior.

To evaluate the contribution in the cyclic voltammograms of the surface capacitive processes and the diffusion-controlled intercalation process, the protocol described in ref. 47. Briefly, it involves using eqn (1):

1 *i*(*V*) = *k*_1_*v* + *k*_2_*v*^1/2^

where *i*(*V*) is the current density at a fixed potential, while *k*_1_*v* and *k*_2_*v*^1/2^ correspond to the weight of the capacitive effect and the interleaving process, where *k*_1_ and *k*_2_ were determined by fitting the dependence with a straight line *i*(*V*)/*v*^1/2^*vs. v*^1/2^.

**Fig. 12:**
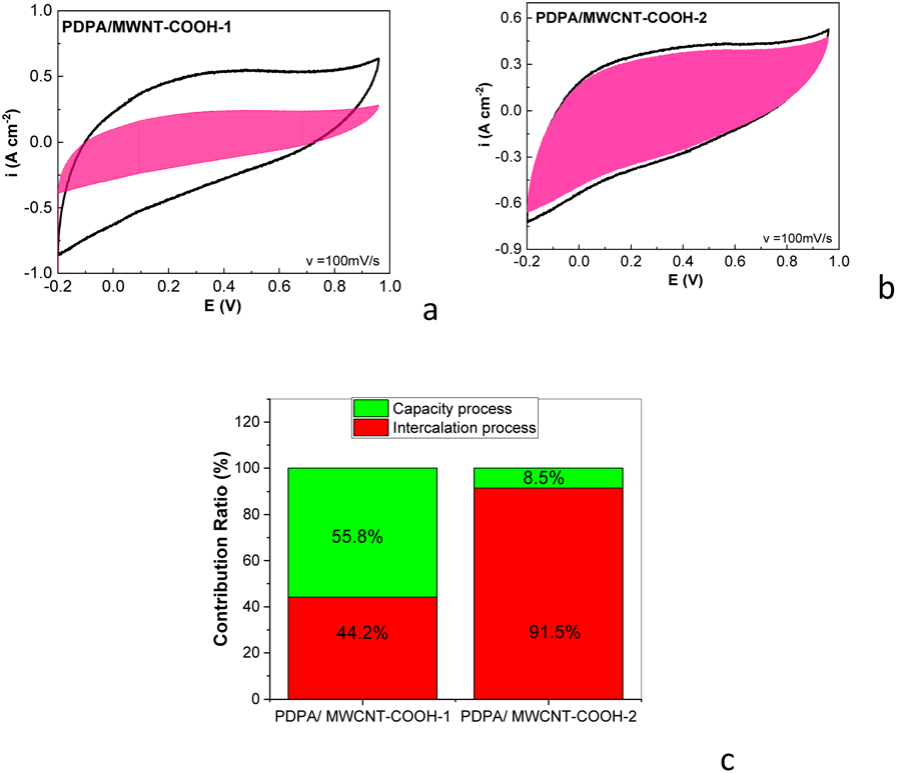
Deconvolution of the intercalation process (magenta area) and capacity (white area) in the case of the supercapacitor cells having electrodes containing the PDPA/MWCNT-COOH-1 (a) and PDPA/MWCNT-COOH-2 (b) composites as active electrode material. Contribution ratio between capacitive and diffusion processes in the case of electrodes containing the PDPA/MWCNT-COOH-1 and PDPA/MWCNT-COOH-2 (c).

The analysis of Fig. 12a–c highlights a more important contribution of the capacitance in the case of supercapacitors containing as active electrode material PDPA/MWCNT-COOH-1 composite in comparison with supercapacitors having as active electrode material PDPA/MWCNT-COOH-2 composite. At the scanning rate of the potential of 100 mV s^−1^, the capacitance of the symmetric supercapacitors having electrodes containing the PDPA/MWCNT-COOH-1 and PDPA/MWCNT-COOH-2 composites as active electrode material is equal to 136.6 mF cm^−2^ and 112.59 mF cm^−2^, respectively, when the concentration of active material in the electrode mass is 80 wt%.

Fig. S1 shows the dependence of capacitance on the concentration of the PDPA/MWCNT-COOH-2 composite in electrode mass, as well as the variation of capacitance of the supercapacitors having the composites PDPA/MWCNT-COOH-1 and PDPA/MWCNT-COOH-2 as electrode active material. Increasing the concentration of PDPA/MWCNT-COOH-2 active material in the electrode mass 80 wt% to 82 wt% and 84 wt% leads to an increase in the capacitance of symmetrical supercapacitors from 112.59 mF cm^−2^ to 129.8 mF cm^−2^ and 145.5 mF cm^−2^, respectively (Fig. S1a). As the potential scan rate decreases from 200 mV s^−1^ to 10 mV s^−1^, it is observed that the capacitance varies in the case of electrodes containing as active material: (a) the PDPA/MWCNT-COOH-1 composite from 81 mF cm^−2^ to 305.17 mF cm^−2^ and (b) the PDPA/MWCNT-COOH-2 composite from 72.76 mF cm^−2^ to 289.65 mF cm^−2^ (Fig. S1b). The lower capacitance values in the case of PDPA/MWCNT-COOH-2, compared to those of PDPA/MWCNT-COOH-1 (Fig. S1b), regardless of the potential scan rate value, can be explained by the defects induced in the carbon nanotubes during the charge/discharge process and the separation process from the spent RLIB cathodes, visualized in the XPS spectra by increasing the weight of C–O–C bonds (Fig. 6g). Considering that the cycle stability of supercapacitors is a key indicator for evaluating their practical application potential, Fig. S2 shows the dependence of capacitance on the number of charge–discharge cycles in the case of PDPA-ES and the PDPA/MWNT, PDPA/MWNT-COOH-1, and PDPA/MWNT-COOH-2 composites. Higher capacitance values of supercapacitors having as active electrode materials PDPA/MWCNT-COOH-1, PDPA/MWCNT-COOH-2 and PDPA/MWNT composites equal to 136.6 mF cm^−2^, 112.59, and 109.82 mF cm^−2^ are reported compared to the capacitance of PDPA equal to 66.13 mF cm^−2^, as a consequence of better ionic diffusion and the presence of more accessible redox sites in the composite materials. The evolution of the capacitance of these supercapacitors, when the voltammograms are recorded at a rate of 100 mV s^−1^, shows a decrease in capacitance as the number of cycles increases. Increasing the number of cyclic voltammograms from 1 to 8000 implies variations in the case of supercapacitors with electrode materials of the following types: (a) PDPA/MWCNT-COOH-1 from 136.6 mF cm^−2^ to 91.23 mF cm^−2^, (b) PDPA/MWCNT-COOH-2, from 112.59 mF cm^−2^ to 83.13 mF cm^−2^, (c) PDPA/MWCNT, from 109.82 mF cm^−2^ to 86.8 mF cm^−2^ and (d) PDPA, from 66.13 mF cm^−2^ to 32.11 mF cm^−2^. Discharge capacity losses of approximately 22%, 16.3%, 20.96% and 51.44% were reported for supercapacitor cells with PDPA/MWCNT-COOH-1, PDPA/MWCNT-COOH-2, PDPA/MWCNT, and PDPA as active electrode materials, respectively. The values of the initial capacitance decrease obtained for the composites prepared by chemical polymerization in the present work are lower than those reported by Aydinli *et al.*^48^ or Parul *et al.*,^49^ which indicates a superior electrochemical stability. This can be associated with a better adhesion of the polymer layer and an increased structural stability. The higher values of supercapacitors with PDPA/MWCNT-COOH-1 and PDPA/MWCNT-COOH-2 composite electrode materials compared to PDPA/MWCNT in the first 3000 cycles can be explained by the presence of carboxyl groups that improve the adhesion of PDPA on the MWCNT surface, thus allowing better access of the electrolyte.

## 4. Conclusions

This study provides the first systematic demonstration of recovering and reusing PDPA/MWCNT-COOH composites from spent RLIB cathodes as active materials for supercapacitor electrodes.

The main conclusions of this article can be highlighted as follows:

(a) The chemical polymerization of DPA in the presence of MWCNT and the oxidant mixture of K_2_Cr_2_O_7_ and H_2_SO_4_ leads to the obtaining of a composite based on PDPA and MWCNT-COOH;

(b) RLIB having as cathode based on the PDPA/MWCNT-COOH-1 composite as active material, discharge capacitance equal to 80 mA h g^−1^ was reported. At EoL, RLIB shows a discharge capacitance equal to 40 mA h g^−1^.

(c) The recovery process of the active material from the cathode of RLIBs involved their collection, disassembly, recovery of the cathode, washing with water in order to eliminate inorganic compounds such as LiPF_6_, Li_2_CO_3_ and Li_2_O, dispersion of the existing compounds in the cathode structure in NMP to eliminate C Super P, interaction of the mixture consisting in PDPA/MWCNT-COOH-1, and PVDF with ethanol to precipitate PVDF and its separation and thus the controlled evaporation of the NMP and C_2_H_5_OH solvents to obtain the PDPA/MWCNT-COOH-2 composite in powder form. Raman scattering, SERS, and FTIR spectroscopy have proved to be important tools in monitoring the separate fractions. The PDPA/MWCNT-COOH-1 and PDPA/MWCNT-COOH-2 composites, prepared from commercial compounds and by recovering from spent RLIB cathodes, contain PDPA in a γ crystalline phase.

(d) The symmetrical supercapacitor cells using the PDPA/MWCNT-COOH-1 and PDPA/MWCNT-COOH-2 composites as electrode materials showed capacitance values equal to 136.6 mF cm^−2^ and 112.59 mF cm^−2^, when the concentration of active material is equal to 80 wt%; the increase of the concentration of the PDPA/MWCNT-COOH-2 composite up to 84 wt% leads to a capacitance of 145.5 mF cm^−2^. These results demonstrate that recovered PDPA/MWCNT-COOH composites not only preserve their structural and electrochemical properties but also show competitive or superior capacitance compared to their commercially synthesized counterparts. The proposed approach offers a sustainable route for reusing RLIB cathodes, contributing to circular economy strategies in advanced energy storage technologies.

Despite the promising results reported in this work, the study presents the following limitations: (i) the electrochemical performance of PDPA/MWCNT-COOH composites recovered from end-of-life lithium-ion batteries was evaluated in symmetric supercapacitor configurations, under laboratory conditions, with cyclic stability tests limited to only 8000 cycles; (ii) the use of aqueous protic electrolytes, which present lower operating voltage and energy density windows; and (ii) the structural and chemical changes induced during cycling and the separation process of lithium rechargeable batteries were qualitatively identified; however, a detailed correlation between defect density and electrochemical performance remains an important aspect to be clarified in the coming period. In this context, future work should aim at: (i) extending the electrochemical evaluation to long-term cycling stability, rate capability and impedance analysis which will be essential to assess the practical applicability of these materials in supercapacitor devices; (ii) the performance of recovered composites should also be investigated in alternative electrolyte configurations and asymmetric or hybrid capacitors to broaden their operating voltage window and energy density; and (iii) the integration of life cycle assessment and techno-economic analysis could clarify the environmental and economic benefits of reusing materials recovered from end-of-life lithium-ion batteries for secondary energy storage applications.

## Conflicts of interest

The authors have no known competing financial interests or personal relationships that could have appeared to influence this manuscript. There are no conflicts to declare.

## Supplementary Material

RA-016-D5RA09202G-s001

## Data Availability

The data supporting this article have been included as part of the supplementary information (SI). Supplementary information is available. See DOI: https://doi.org/10.1039/d5ra09202g.
