# Supplementary material for: Comparative analysis of polydiphenylamine/MWCNT-COOH composites for supercapacitors: recovered vs. commercial nanotube electrodes
Source: RSC Adv. 2026 Mar 4;16(14):12302–14. doi: 10.1039/d5ra09202g (PMC12958025; doi:10.1039/d5ra09202g)
Supplement: RA-016-D5RA09202G-s001 [file RA-016-D5RA09202G-s001.pdf]

Supplementary Information (SI) for RSC Advances.
This journal is © The Royal Society of Chemistry 2026

Supporting Information

Comparative Analysis of Polydiphenylamine/MWCNT Composites for Supercapacitors: Recovered vs. Commercial Nanotube Electrodes

C.S. Florica[a,b], A. Nila[b], M. Vaduva[b], C. Negrila[b], C. Bartha[b], Marouane Aannir[c], I. Saadoune[c], S. Bellucci[b,d,e], O. Cramariuc[f] and M. Baibarac[b*]

[a] *University of Bucharest, Faculty of Physics, Atomistilor Street 405, Magurele, Romania.*

[b] *National Institute of Materials Physics, Laboratory of Optical Processes in Nanostructured Materials, Atomistilor street 405A, Magurele, Romania*

[c] *Mohammed VI Polytech Univ UM6P, ACER, Lot 660, Hay Moulay Rachid, Ben Guerir 43150, Morocco.*

[d] *Nano Research Laboratory, Excellent Center, Baku State University, Baku, Azerbaijan.*

[e] *Ecotec University, Samborondón, Ecuador.*

[f] IT Ctr Sci & Technol, 25 Av Radu Beller Str, Bucharest 011702, Romania.

[*]Corresponding author: Mihaela Baibarac

E-mail address: barac@infim.ro

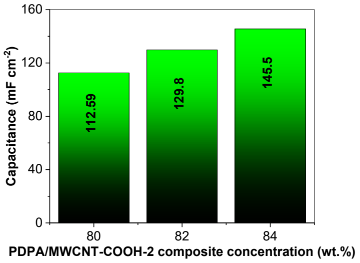

a

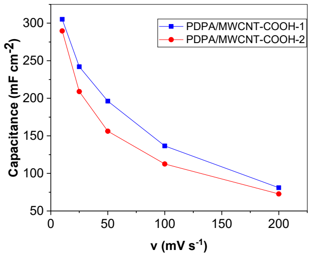

b

Figure S1. Dependence of the capacitance of symmetrical supercapacitors having electrodes based on the PDPA/MWCNT-COOH-2 composite as a function of the concentration of active material (a). The dependence of capacitance of supercapacitors having the composites PDPA/MWCNT-COOH-1 and PDPA/MWCNT-COOH-2 as electrode active material (concentration of active material is 80 wt.%) (b).

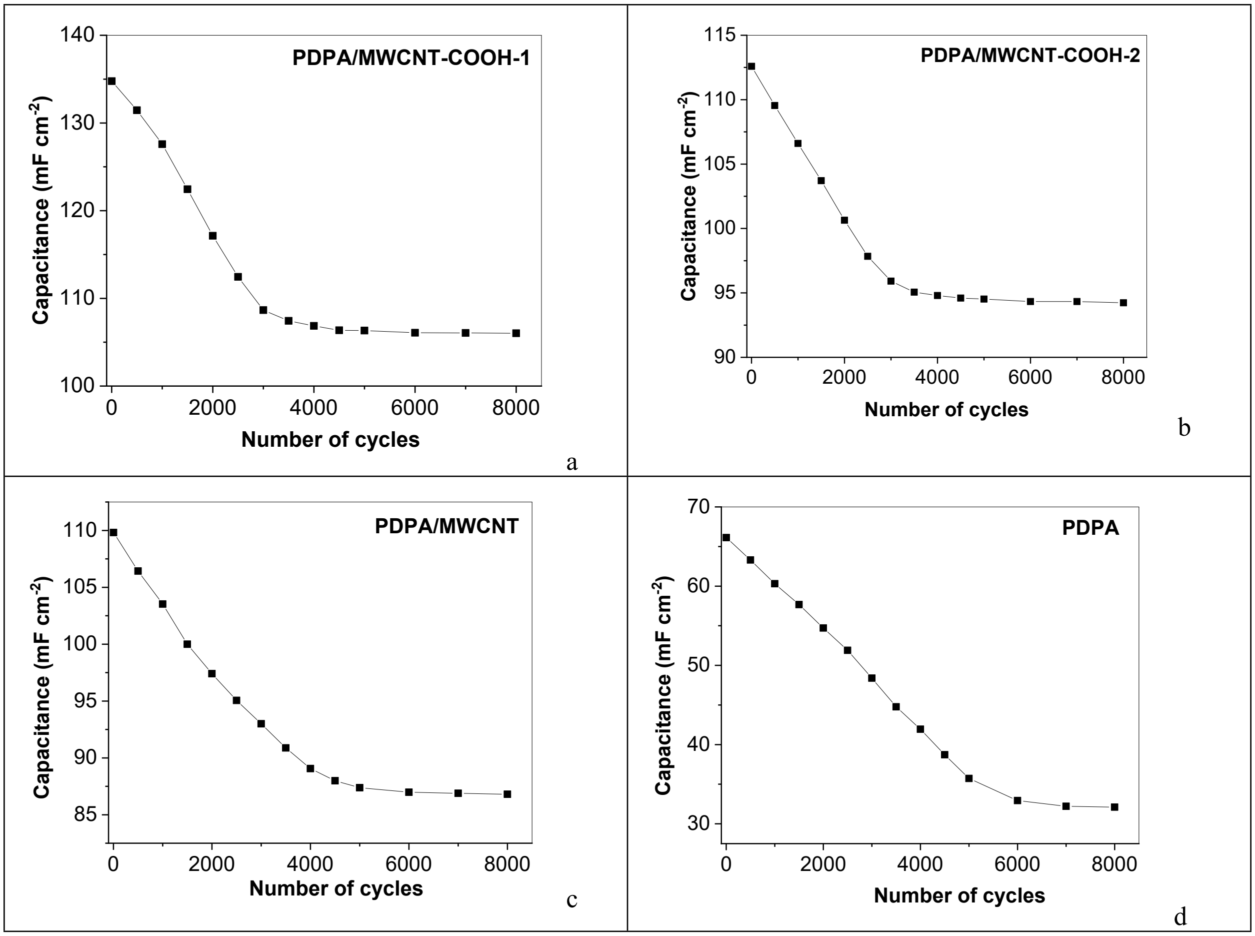

Figure 2. The dependence of capacitance of the symmetrical supercapacitors having as electrode active materials: a) PDPA/MWCNT-COOH-1, b) PDPA/MWCNT-COOH-1, c) PDPA/MWCNT, and d) PDPA. The concentration of active material in the electrode's weight is 80 wt.%.
